# Mediating role of systemic inflammation in linking transferrin saturation to all-cause mortality in patients with coronary artery disease: Evidence from a large population-based study

**DOI:** 10.1371/journal.pone.0322633

**Published:** 2025-06-02

**Authors:** Zhenzhen Chen, Juan Guo, Haoying Chen, Yingying Guan, Simin Wang, Jianyu Zhou

**Affiliations:** 1 Department of Ultrasound, Taizhou Central Hospital, Taizhou, Zhejiang Province, China; 2 Clinical Education Team, GE HealthCare Ultrasound, Wuhan, Hubei Province, China; University of Diyala College of Medicine, UNITED STATES OF AMERICA

## Abstract

**Background:**

Transferrin saturation (TS) is associated with mortality across populations, but its nonlinear relationship with all-cause mortality in coronary artery disease (CAD) and the role of systemic inflammation remain unclear. This study explored the association between TS and mortality in CAD patients, focusing on systemic inflammation as a potential mediator.

**Methods:**

Data from National Health and Nutrition Examination Survey (NHANES) 1999–2006 included 769 CAD patients (>18 years) with available TS and mortality records. Systemic inflammation markers, such as the systemic immune-inflammation index (SII) and systemic inflammation response index (SIRI), were analyzed. Kaplan–Meier curves, Cox proportional hazards models, and mediation analyses examined the interactions between TS, inflammation, and mortality.

**Results:**

A U-shaped relationship between TS and all-cause mortality was observed, with an inflection point at 30.5%. TS levels ≤30.5% were inversely associated with mortality (HR = 0.98; 95% CI, 0.96–0.99; P < 0.0001), while levels >30.5% increased mortality risk (HR = 1.05; 95% CI, 1.02–1.08; P < 0.001). Systemic inflammation markers (SII/SIRI) were associated with and may partially mediate the relationship between low TS (≤30.5%) and mortality. (mediation proportions: 28.5% and 21.8%, respectively). No mediation effects were found for TS > 30.5%.

**Conclusions:**

TS demonstrates a U-shaped relationship with all-cause mortality in CAD patients. Systemic inflammation is linked to both TS and mortality outcomes, suggesting potential mechanistic interplay. Maintaining TS within 20–30% and addressing inflammation may reduce mortality risk.

## Introduction

Iron metabolism and systemic inflammation are increasingly recognized as critical factors influencing mortality, particularly among individuals with cardiovascular diseases [1–3]. Transferrin saturation(TS), a key biomarker of iron metabolism, has been shown to exhibit a non-linear relationship with mortality, where both low and high levels are associated with adverse outcomes [4,5]. Mechanistically, chronic inflammation triggers interleukin-6 (IL-6) secretion, which upregulates hepcidin — the master regulator of iron homeostasis [6]. Elevated hepcidin inhibits intestinal iron absorption and macrophage iron recycling, leading to functional iron deficiency despite normal iron stores (low TS) [7]. Conversely, iron overload (high TS) promotes ferroptosis, an iron-dependent form of cell death driven by lipid peroxidation and glutathione depletion, which exacerbates cardiomyocyte apoptosis and plaque instability. However, the mechanisms underlying this association remain poorly understood, especially in high-risk populations such as patients with coronary artery disease (CAD). Existing studies have primarily concentrated on the direct relationship between TS and mortality in general populations, with limited exploration of the mediating role of systemic inflammation [8–10]. This gap is significant, as inflammation is a hallmark of CAD and is closely linked to both iron metabolism dysregulation and mortality risk

Systemic inflammation, as indicated by markers such as neutrophil-to-lymphocyte ratio (NLR), immune-inflammation index (SII), and C-reactive protein (CRP), has been shown to independently predict mortality in various populations [11–13]. Inflammation can disrupt iron homeostasis by altering iron transport and storage, leading to functional iron deficiency, even in the presence of adequate iron stores [6,7]. Conversely, iron dysregulation can exacerbate inflammation through oxidative stress and immune activation, creating a bidirectional relationship [14,15]. Despite these known interactions, few studies have systematically evaluated the mediating role of inflammation in the relationship between TS and mortality, particularly in CAD patients. This is a critical gap, as CAD patients often exhibit heightened inflammatory states, which may amplify the impact of iron metabolism abnormalities on mortality.

Previous research has demonstrated significant associations between iron biomarkers, such as serum ferritin and TS, with all-cause and cardiovascular mortality [16,17]. However, findings have been inconsistent, with some studies reporting J-shaped or linear associations, while others found no significant relationships [18]. These discrepancies may stem from differences in study populations, methodologies, and the lack of consideration for potential mediators such as inflammation. For instance, studies in diabetic and non-diabetic populations have highlighted distinct patterns in the association between iron biomarkers and mortality [16,19], suggesting that comorbidities and inflammatory states may modify these relationships. Furthermore, while inflammation has been implicated as a key factor in iron metabolism and mortality, its role as a mediator has not been adequately quantified in prior studies.

This study addresses these gaps by investigating the mediating role of systemic inflammation in the relationship between TS and all-cause mortality in CAD patients, using data from the National Health and Nutrition Examination Survey (NHANES) database. By incorporating advanced statistical methods, this study provides a nuanced understanding of the complex interplay between iron metabolism, inflammation, and mortality. The findings have the potential to clarify the mechanisms underlying TS-mortality associations and to inform targeted interventions aimed at reducing mortality risk in CAD patients. This approach not only builds on existing evidence but also introduces a novel perspective by emphasizing the importance of systemic inflammation as a mediator, thereby advancing the understanding of iron metabolism in the context of cardiovascular disease.

## Materials and methods

### Study population

This longitudinal cohort study utilized data from NHANES, a nationwide survey designed to assess the health status of the U.S. population. Through a stratified multistage random sampling approach, NHANES ensures nationally representative data. Ethical approval was obtained from the National Center for Health Statistics’ review board, and all participants provided informed consent. The datasets, along with detailed documentation and standardized protocols, are publicly available on the NHANES website, consistent with the methodologies used in previous studies.

**Fig 1:**
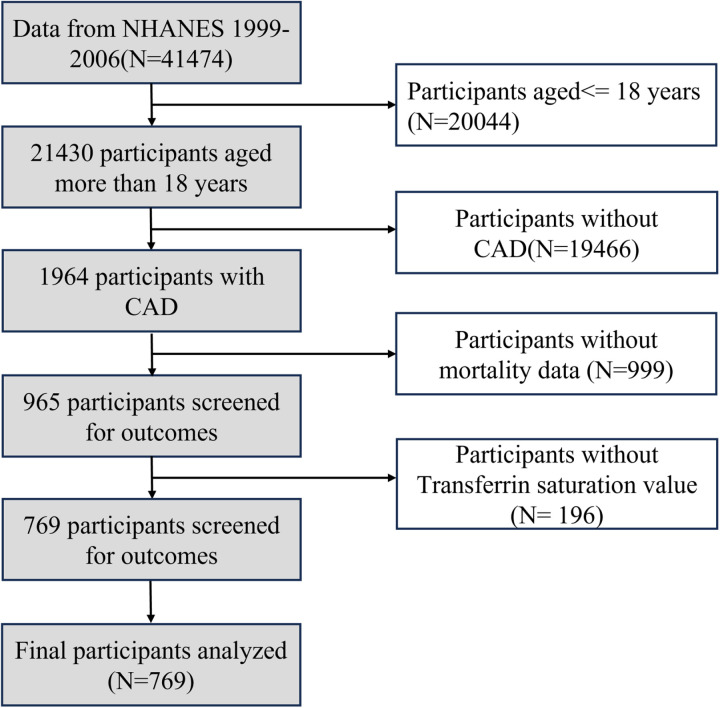
Flowchart of study participants.

Data from the 1999–2006 NHANES cohort (N = 41,474) were analyzed. Participants aged ≤18 years (N = 20,044) were excluded. Among the remaining 21,430 participants aged >18 years, those without CAD (N = 19,466) were excluded. Of the 1,964 participants with CAD, individuals without mortality data (N = 999) and those lacking TS values (N = 196) were further excluded. Ultimately, 769 participants were included in the final analysis (Fig 1).

### Exposure variable and outcomes

The exposure variable, TS (%), was calculated as serum iron divided by total iron-binding capacity (TIBC) × 100%. Serum iron was measured using a timed-endpoint method, and TIBC was calculated as the sum of serum iron and unsaturated iron-binding capacity (UIBC). All measurements were performed using a Beckman/Coulter LX20 analyzer. Detailed methods are available at http://cdc.gov/nchs/nhanes.

The outcome variable was all-cause mortality among participants with CAD. Mortality data were obtained from the National Center for Health Statistics by linking NHANES data to death certificate records in the National Death Index using a validated matching method. Mortality follow-up data were available from the date of survey participation until December 31, 2006. Participants not matched to any death records were considered alive through the follow-up period. We assessed all-cause mortality, with causes of death determined using the International Classification of Diseases and Related Health Problems, Tenth Revision (ICD-10).

### Covariates

In the present study, the following covariates were collected and analyzed: gender, age, race, education level, family poverty-to-income ratio (PIR), body mass index (BMI), diabetes mellitus (DM), hypertension, hemoglobin, triglycerides (TG), low-density lipoprotein cholesterol (LDL-C), monocyte count, neutrophil count, lymphocyte count, platelet count, SII), SIRI, NLR, platelet-to-lymphocyte ratio (PLR), monocyte-to-lymphocyte ratio (MLR), CRP, Additionally, follow-up time and mortality status were recorded. The self-reported questionnaires were used for the diagnoses of hypertension (BPD035), DM (DIQ010), CAD(MCQ160c).

BMI was calculated as the ratio of weight (kg) to height (m) squared. Race was categorized into five groups: Non-Hispanic White, Non-Hispanic Black, Mexican American, Other Hispanic, and Other Race. Education level was stratified into three categories: below high school, high school, and above high school. Family PIR was used as an indicator of socioeconomic status. Hypertension and DM were defined based on self-reported diagnoses. Inflammatory markers, including SII, SIRI, NLR, PLR, and MLR, were calculated as follows:

SII = Platelet count × neutrophil count/ lymphocyte count

SIRI = Neutrophil count × monocyte count/ lymphocyte count

NLR = Neutrophil count/ lymphocyte count

PLR = Platelet count/ lymphocyte count

MLR = Monocyte count/ lymphocyte count

### Statistical analysis

All statistical analyses were performed using R software (version 4.2.2), EmpowerStats (version 2.0), the rms package, and MSTATA. Categorical variables were summarized as counts and proportions, while continuous variables were described using means ± standard deviations or medians with interquartile ranges, depending on their distributions. The Chi-square test or Kruskal-Wallis H test was employed to evaluate differences among tertiles of TS. Statistical significance was set at a two-sided p-value of less than 0.05.

Missing covariates were addressed through multiple imputation by chained equations (MICE) using 5 imputed datasets. Convergence was verified via trace plots over 20 iterations. Complete case analysis (n = 689) yielded consistent results (ΔHR < 5% for primary outcomes, see S1 File).

Multivariate Cox proportional hazard models were performed to explore the associations between TS, systemic inflammation markers, and all-cause mortality. The systemic inflammation markers included the SII, SIRI, MLR, NLR, PLR and NPR. The results were presented as hazard ratios (HRs) with 95% confidence intervals (CIs) across three models. Model 1 was unadjusted. Model 2 adjusted for gender and age. Model 3 further adjusted for gender, age, race, PIR, DM, hypertension, triglycerides, CRP, HDL-C, and hemoglobin.

The nonlinear TS-mortality relationship was characterized through sequential analysis: (1) Preliminary Identification: Penalized splines (R package mgcv) revealed significant deviation from linearity;(2) Segmented Modeling: Cox proportional hazards models with iterative grid search (5th-95th TS percentile, 5% increments) identified 30.5% as optimal inflection point;(3) Threshold Validation: Log-likelihood ratio test confirmed superior fit of threshold model vs. linear model, with <1% deviation in sensitivity analyses using restricted cubic splines (R package rms).Kaplan–Meier survival curves were plotted to visualize the survival rates of participants across tertiles of TS for all-cause mortality. Log-rank tests were used to compare differences in survival between groups.

Mediation analysis was conducted using the “mediation” package in R 4.2.2 to evaluate the mediating effects of systemic inflammation-related indicators (IRIs): SII, SIRI, NLR, and CRP on the associations between TS and all-cause mortality. The analysis was adjusted for gender, age, race, poverty-to-income ratio (PIR), diabetes mellitus (DM), hypertension, triglycerides, CRP, high-density lipoprotein cholesterol (HDL-C), and hemoglobin in Model 3.A mediating effect was identified when the following conditions were met: a significant indirect effect, a significant total effect, and a positive proportion of the mediating effect. Subgroup analyses were conducted to investigate the relationships between TS and all-cause mortality across different populations, including subgroups stratified by gender, race, education level, family PIR, BMI, hypertension, diabetes, smoking.

E-value analysis was implemented to quantify the minimum strength of association required between unmeasured confounders and both the exposure (TS) and outcome (mortality) to negate the observed mediation effects. Reverse mediation models were constructed by interchanging the mediator (inflammatory markers) and exposure (TS), with inverse probability weighting to test for bidirectional pathway significance.

### Ethics approval and consent to participate

The NHANES study was approved by the Ethics Review Board of the National Center for Health Statistics, and all participants provided informed written consent at the time of enrollment.

## Results

### Participants characteristics

Table 1 summarizes the baseline characteristics of participants stratified by tertiles of TS. Across all participants, TS ranged from 12.69 ± 3.50 in T1, 21.33 ± 2.49 in T2, to 34.38 ± 8.92 in T3(P ＜ 0.001). Compared to participants in the lower tertiles of TS, those in the higher tertiles had a greater proportion of males and significantly higher family PIR levels. Participants in higher tertiles also exhibited lower BMI, platelet count, neutrophil count, and CRP levels, along with improved albumin and hemoglobin concentrations. Additionally, systemic inflammation markers such as SII, SIRI, NLR, and PLR demonstrated a decreasing trend across the tertiles, while NPR remained consistent. Furthermore, participants with higher TS were less likely to have hypertension and showed lower levels of WBC and triglycerides.

**Table 1:** Characteristics of the study population with various TS tertiles.

Characteristics	Tertiles of transferrin saturation			*P* value
	T1(n = 215)	T2(n = 268)	T3(n = 286)	
**Gender**				<0.001
Female	111 (51.63%)	108 (40.30%)	95 (33.22%)	
Male	104 (48.37%)	160 (59.70%)	191 (66.78%)	
**Age (years)**				
>18, <=45	12 (5.58%)	18 (6.72%)	17 (5.94%)	0.865
>45, <=65	69 (32.09%)	82 (30.60%)	100 (34.97%)	0.538
>65	34 (62.33%)	168 (62.69%)	169 (59.09%)	0.638
**BMI, kg/m** ^**2**^	30.00 ± 7.49	29.29 ± 5.69	28.13 ± 5.41	0.004
**Race, n (%)**				0.026
Non-Hispanic Black	49 (22.79%)	49 (18.28%)	32 (11.19%)	
Mexican American	40 (18.60%)	43 (16.04%)	48 (16.78%)	
Other Hispanic	8 (3.72%)	9 (3.36%)	11 (3.85%)	
Other Race	5 (2.33%)	2 (0.75%)	8 (2.80%)	
Non-Hispanic White	113 (52.56%)	165 (61.57%)	187 (65.38%)	
**Education level, n (%)**				0.367
Below high school	97 (45.5%)	112 (41.8%)	127 (44.6%)	
High school	55 (25.8%)	66 (24.6%)	56 (19.6%)	
Above high school	61 (28.6%)	90 (33.6%)	102 (35.8%)	
**Family PIR**	2.05 ± 1.39	2.46 ± 1.60	2.49 ± 1.61	0.005
**DM, n (%)**	68 (32.08%)	68 (25.86%)	83 (29.75%)	0.315
**Hypertension, n (%)**	154 (71.63%)	172 (64.18%)	175 (61.19%)	0.048
**Monocyte, 10** ^**9**^ **/L**	0.64 ± 0.22	0.61 ± 0.19	0.62 ± 0.26	0.242
**Platelet,10** ^**9**^ **/L**	270.99 ± 88.78	250.73 ± 66.12	236.55 ± 67.30	<0.001
**Neutrophils, 10** ^**9**^ **/L**	4.90 ± 1.89	4.41 ± 1.59	4.23 ± 1.43	<0.001
**Lymphocyte,10** ^**9**^ **/L**	2.07 ± 1.16	1.96 ± 0.76	2.01 ± 0.78	0.394
**SII**	641.41 (428.56-865.35)	524.86 (374.66-761.63)	472.22 (357.00-672.77)	<0.001
**SIRI**	1.86 ± 1.56	1.54 ± 0.89	1.48 ± 0.97	<0.001
**MLR**	0.36 ± 0.18	0.35 ± 0.15	0.34 ± 0.15	0.310
**NLR**	2.96 ± 2.38	2.59 ± 1.44	2.37 ± 1.20	<0.001
**PLR**	155.37 ± 91.00	144.87 ± 66.22	131.85 ± 59.53	0.001
**NPR**	0.02 ± 0.02	0.02 ± 0.03	0.02 ± 0.01	0.864
**CRP (mg/dL)**	0.56 (0.22-1.30)	0.33 (0.16-0.69)	0.23 (0.10-0.48)	<0.001
**Hemoglobin (g/dL)**	13.39 ± 1.68	14.11 ± 1.43	14.51 ± 1.50	<0.001
**TG (mg/dL)**	182.25 ± 201.56	162.82 ± 104.93	170.89 ± 165.11	0.418
**LDL-C (mg/dL)**	111.91 ± 37.43	120.04 ± 41.99	115.27 ± 34.56	0.396
**TS (%)**	12.69 ± 3.50	21.33 ± 2.49	34.38 ± 8.92	<0.001
**Follow-up time(months)**	57.82 ± 23.53	60.99 ± 19.67	63.82 ± 19.35	0.006
**Mortality, n (%)**	73 (33.95%)	77 (28.73%)	73 (25.52%)	0.119

BMI: Body Mass Index; PIR: Poverty Income Ratio; DM: Diabetes Mellitus; SII: Systemic Immune-Inflammation Index; SIRI: Systemic Inflammation Response Index; MLR: Monocyte-to-Lymphocyte Ratio; NLR: Neutrophil-to-Lymphocyte Ratio; PLR: Platelet-to-Lymphocyte Ratio; NPR: Neutrophil-to-Platelet Ratio; TC: Total Cholesterol; TG: Triglycerides; TS: Transferrin saturation; LDL-C: Low-Density Lipoprotein Cholesterol; HDL-C: High-Density Lipoprotein Cholesterol; CRP: C-Reactive Protein;

### Nonlinear association between TS and coronary heart disease all-cause mortality

**Fig 2:**
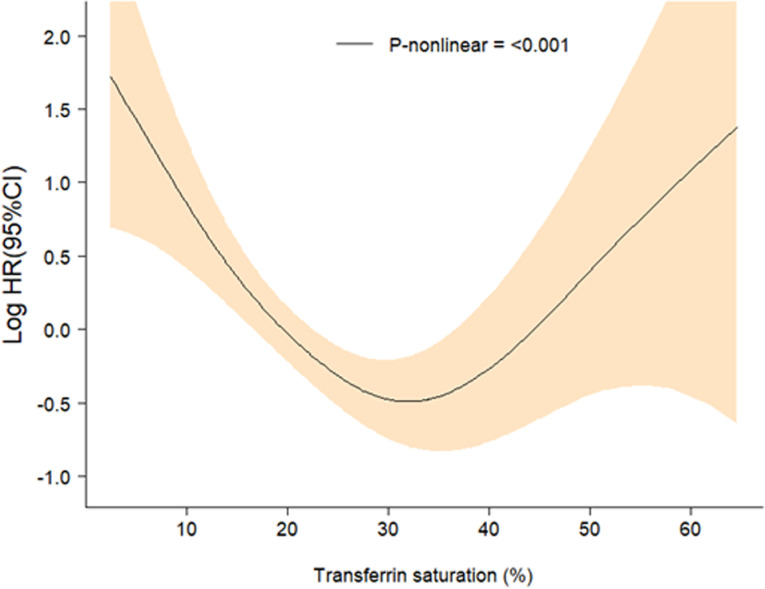
Relationship between TS and all-cause mortality. Generalized additive models revealed significant nonlinear associations between TS and mortality (P < 0.001). Solid lines represent the estimated hazard ratios, and shaded areas denote 95% confidence intervals. adjustments were made for gender, age, race, BMI, DM, hypertension, triglyceride, CRP, HDL, Hemoglobin.

To investigate the nonlinear relationship between TS and CAD mortality, we utilized Cox proportional hazards regression models, generalized additive models, and smooth curve fitting employing penalized splines. Fully adjusted smooth curve fitting analyses revealed significant nonlinear associations between TS and CAD mortality (Fig 2).

To further explore the threshold effects of TS on all-cause mortality in CAD, we conducted analyses using both the standard Cox proportional hazards model and Segmented regression (Table 2). The log-likelihood ratio test indicated P values <0.01, suggesting that the two-piecewise Cox proportional hazards model better captured the relationship between TS and mortality.

**Table 2:** Threshold effect analysis of TS on mortality.

All mortality	HR (95 CI%)		
	Model 1	Model 2	Model 3
Transferrin saturation (%)	1.00 (0.99, 1.01)	1.00 (0.99, 1.00)	0.99 (0.99, 1.00)
Inflection point			
≤30.5	0.98 (0.97, 1.00)	0.98 (0.96, 0.99)	0.97 (0.95, 0.99)
>30.5	1.03 (1.01, 1.06)	1.04 (1.02, 1.06)	1.05 (1.02, 1.08)
Log likelihood ratio	<0.001	<0.001	<0.001

Model 1: adjust for: none. Model 2 adjust for: gender, age. Model 3 adjust for: gender, age, race, BMI, smoking, DM, hypertension, triglyceride, CRP, LDL, Hemoglobin

As shown in Table 2, non-linear associations were observed between TS and all-cause and CAD mortality (P for the log-likelihood ratio < 0.05). After adjusting for covariates, the inflection point for TS was identified as 30.5%. When TS was below 30.5%, increases in TS were associated with a significantly reduced risk of all-cause mortality (HR = 0.97; 95% CI, 0.96–0.99; P < 0.0001). However, when TS exceeded 30.5%, higher TS levels showed significantly increased risks for all mortality (HR = 1.05; 95% CI, 1.02–1.08; P < 0.001). These findings suggest a complex dose-response relationship between TS and mortality risks that is highly dependent on the identified inflection point.

### Kaplan–Meier curves of survival rates by TS tertiles

**Fig 3:**
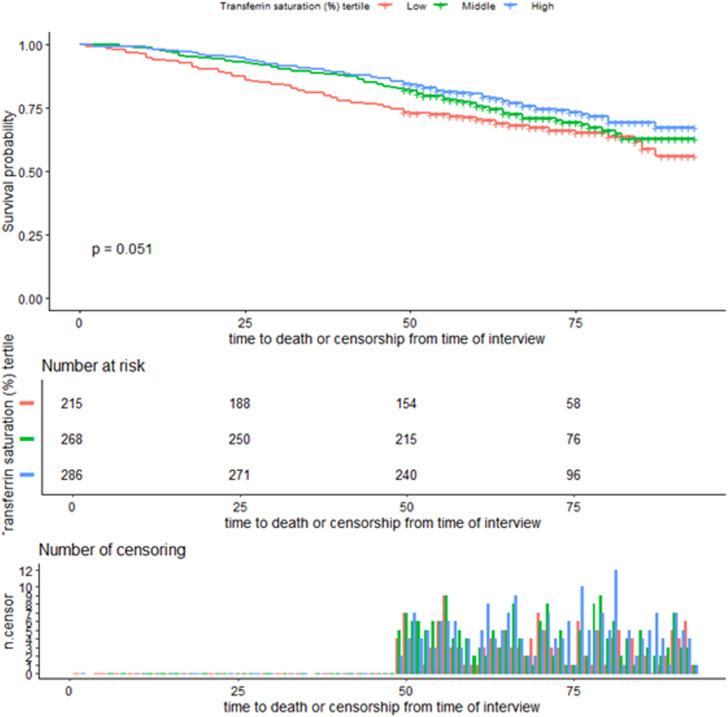
Kaplan–Meier curves of the survival rate of participants with TS tertiles and all-cause mortality.

Kaplan-Meier survival analysis showed lower survival probabilities in the low TS group compared to the middle and high TS groups throughout the follow-up period (Fig 3). By the 75th month, survival probabilities were 55.7%, 62.6%, and 67.6% for the low, middle, and high TS groups, respectively. Log-rank test showed a near-significant difference among the three groups (P = 0.051). Although the low TS group exhibited the lowest survival, the difference did not reach statistical significance. Censoring was distributed evenly across the groups.

### Subgroup analysis

**Fig 4:**
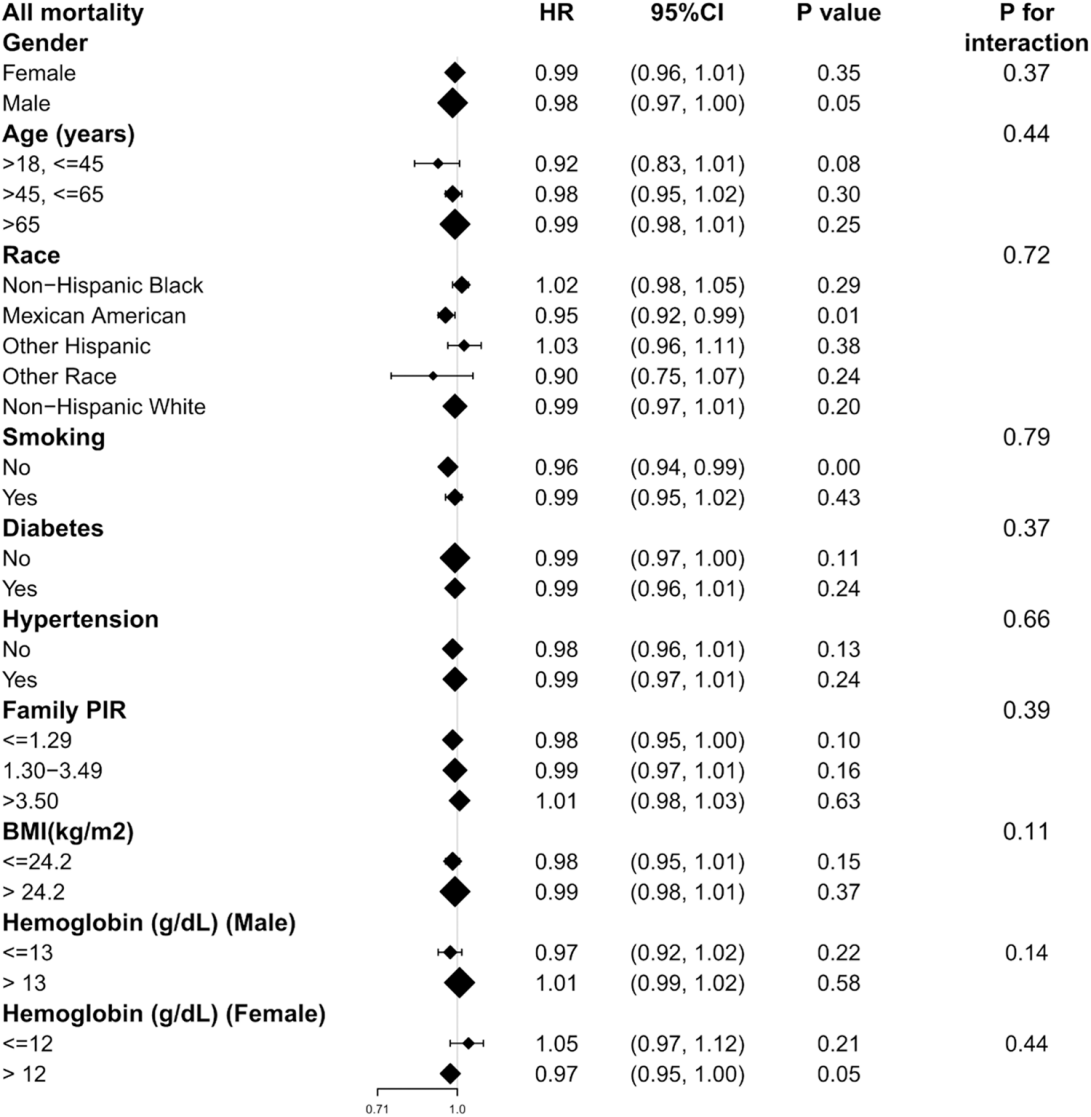
Subgroup analysis of associations between TS and all mortality.

Subgroup analyses were conducted to examine the associations of TS with all-cause mortality (Fig 4). Across all subgroup analyses, no statistically significant associations or interactions were identified in any examined strata (P for interaction > 0.05 for all comparisons), indicating a consistent lack of evidence for subgroup-specific differences.

### Associations of TS with IRIs and mortality

Table 3 shows the associations between TS and IRIs across three models. In the fully adjusted Model 3, TS was significantly and negatively associated with most IRIs, including SII (β = -0.0043, 95% CI = -0.0049 to -0.0036, P < 0.001), NLR (β = -1.07, 95% CI = -1.28 to -0.85, P < 0.001), PLR (β = -0.02, 95% CI = -0.02 to -0.01, P < 0.001), SIRI (β = -1.44, 95% CI = -1.72 to -1.17, P < 0.001), and MLR (β = -5.54, 95% CI = -7.80 to -3.29, P < 0.001). Similarly, NPR was negatively associated with TS in the fully adjusted model (β = -68.95, 95% CI = -98.12 to -39.78, P < 0.001). However, MLR showed no significant association with TS in Model 1 (P = 0.19), and the relationship between NPR and TS was non-significant in Model 1 as well (P = 0.088).

**Table 3:** Associations between TS and inflammatory markers.

IRIs	HR (95 CI%), *P*-value		
	Model 1	Model 2	Model 3
SII	−0.0035 (−0.0039, −0.0032) <0.001	0.0040 (−0.0044, −0.0036) <0.001	0.0043 (−0.0049, −0.0036) <0.001
MLR	−0.80 (−1.98,0.38) 0.19	−6.45 (−7.65, −5.25) <0.001	−5.54 (−7.80, −3.29) <0.001
NLR	−0.49 (−0.61, −0.37) <0.001	−0.85 (−0.97, −0.73) <0.001	−1.07 (−1.28, −0.85) <0.001
PLR	−0.009 (−0.012, −0.006) <0.001	−0.01 (−0.014, −0.008) <0.001	−0.02 (−0.02, −0.01) <0.001
SIRI	−1.02 (−1.17, −0.87) <0.001	−1.45 (−1.60, −1.30) <0.001	−1.44 (−1.72, −1.17) <0.001
NPR	−8.05 (−17.33, 1.23) 0.089	−20.29 (−29.44, −11.14) <0.001	−68.95 (−98.12, −39.78) <0.001

Model 1: adjust for: none. Model 2 adjust for: gender, age. Model 3 adjust for: gender, age, race, BMI, DM, hypertension, triglyceride, CRP, HDL, Hemoglobin

Table 4 presents the associations between IRIs and all-cause mortality based on multivariate Cox proportional hazard models. The fully adjusted Model 3 demonstrated that higher levels of SII (HR = 1.0003, 95% CI = 1.0002–1.0004, P < 0.0001), MLR (HR = 3.68, 95% CI = 2.29–5.90, P < 0.0001), NLR (HR = 1.19, 95% CI = 1.13–1.24, P < 0.0001), PLR (HR = 1.003, 95% CI = 1.001–1.004, P < 0.0001), and SIRI (HR = 1.20, 95% CI = 1.12–1.28, P < 0.0001) were significantly associated with an increased risk of all-cause mortality. However, NPR did not show a significant association with all-cause mortality in Model 3 (P = 0.12), although it demonstrated a strong association in Model 1 (P < 0.0001) and Model 2 (P < 0.0001).

**Table 4:** The associations of inflammatory markers with all-cause mortality.

IRIs	HR 95%CI, *P*-value		
	Model 1	Model 2	Model 3
SII	1.0005 (1.0004,1.0006) <0.0001	1.0004 (1.0003,1.0004) <0.0001	1.0003 (1.0002,1.0004) <0.0001
MLR	11.12 (8.60,14.37) <0.0001	3.23 (2.24,4.64) <0.0001	3.68 (2.29, 5.90) <0.0001
NLR	1.25 (1.21,1.29) <0.0001	1.16 (1.13,1.20) <0.0001	1.19 (1.13, 1.24) <0.0001
PLR	1.004 (1.0028,1.0047) <0.0001	1.002 (1.001,1.003) <0.0001	1.003 (1.001, 1.004) <0.0001
SIRI	1.24 (1.20,1.28) <0.0001	1.18 (1.13,1.22) <0.0001	1.1979 (1.12, 1.28) <0.0001
NPR	463.02 (51.23,4185.04) <0.0001	547.29 (39.98,7492.46) <0.0001	81.61 (0.32, 21028.17) 0.12

Model 1: adjust for: none. Model 2 adjust for: gender, age. Model 3 adjust for: gender, age, race, BMI, DM, hypertension, triglyceride, CRP, HDL, Hemoglobin

### Mediating role of IRIs

**Fig 5:**
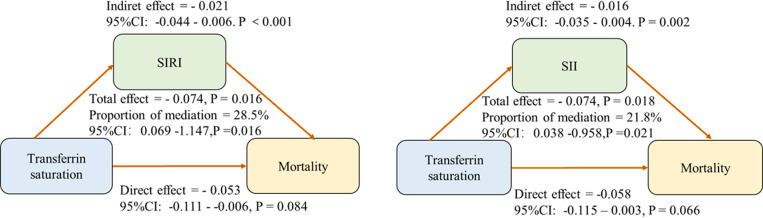
Analysis of the mediation by inflammatory markers of the associations between TS and mortality when TS ≤ 30.5%.

Fig 5, Table 5 shows that SIRI mediated 21.8% of the association between transferrin saturation (TS) and all-cause mortality, while SII mediated 28.5% of the association. The mediating effects of these systemic inflammatory markers highlight their significant role in the relationship between TS and mortality. Additional analyses of other inflammatory indicators, including NLR and PLR, are shown in S1 Table in S1 File. S2 Table in S1 File displays the reverse mediation analysis in patients with TS ≤ 30.5%, with inflammatory markers demonstrating mediation proportions of 6.2% for SIRI (P = 0.214) and 7.8% for SII (P = 0.199). S3 Table in S1 File presents the mediation analysis when TS > 30.5%, showing SIRI accounted for 1.3% (P = 0.806) and SII accounted for 2.8% (P = 0.753) of the association between TS and mortality.

**Table 5:** Mediation effects of inflammatory markers on the association between TS and mortality when TS ≤ 30.5%.

IRIs	Proportion of mediation	95% CI lower	95% CI upper	P-value
SIRI	0.285	0.069	1.147	0.016
SII	0.218	0.038	0.958	0.021
MLR	0.130	−0.053	0.584	0.101
NLR	0.174	−0.023	0.696	0.072
PLR	0.077	−0.015	0.411	0.096
CRP	0.168	−0.009	0.593	0.064
NPR	0.015	−0.028	0.427	0.460

## Discussion

Our study revealed a nonlinear relationship between TS and all-cause mortality in CAD patients, with a clear inflection point at 30.5%. Below this threshold, TS was inversely associated with mortality, while levels above it showed a significant positive association with mortality. This U-shaped pattern corroborates previous evidence that extremes of iron status may have detrimental health effects [18]. Importantly, systemic inflammatory markers, such as SIRI and SII, demonstrated differential mediating effects between TS and mortality depending on the TS level. These findings suggest that the underlying pathophysiological mechanisms driving mortality vary according to TS status, with inflammation playing a central role at lower levels of TS and oxidative stress and iron overload toxicity dominating at higher levels.

In the TS ≤ 30.5% range, mortality risk appears to be predominantly driven by iron deficiency and its downstream effects on systemic inflammation. Iron deficiency leads to significant disruptions in immune function, particularly by promoting the activation of neutrophils and monocytes, which produce pro-inflammatory cytokines and exacerbate systemic inflammation [20]. In this study, SIRI mediated 28.5% and SII mediated 21.8% of the TS–mortality relationship in individuals with low TS, reflecting the prominent role of inflammation in this context. Furthermore, insufficient iron limits erythropoiesis and oxygen-carrying capacity, resulting in tissue hypoxia that activates reactive oxygen species (ROS) generation and inflammatory pathways, further aggravating cardiovascular stress. These combined mechanisms link iron deficiency with heightened mortality risk, particularly in CAD patients who are already vulnerable to ischemic injury and systemic inflammation.

Conversely, in the TS > 30.5% range, the mediating role of inflammation is markedly diminished, with SIRI accounting for only 1.3% of the observed association between TS and mortality (P = 0.806). Instead, iron overload at higher TS levels appears to drive mortality risk through direct toxic effects. Elevated TS reflects increased levels of non-transferrin-bound iron, which catalyzes the formation of ROS via the Fenton reaction [21]. This promotes oxidative stress, lipid peroxidation, and endothelial dysfunction, accelerating atherosclerosis, plaque destabilization, and thrombosis [21,22]. Hyperferritinemia, a marker of chronic inflammation often associated with iron overload, can further exacerbate oxidative damage and metabolic dysfunction [23,24]. These findings suggest that at higher TS levels, mortality risk is primarily attributable to iron-induced oxidative damage and organ dysfunction rather than systemic inflammation, highlighting the stark shift in the underlying pathology as TS surpasses the 30.5% threshold.

From a clinical perspective, these results highlight the dual risks posed by TS extremes and underscore the importance of maintaining TS within an optimal range of 20–30% For patients with low TS (≤30.5%), particularly those at highest risk (TS < 15%), we recommend addressing both iron deficiency and inflammation concurrently. These patients should undergo iron status evaluation, especially when presenting with anemia or fatigue. The IRONMAN trial (EJHF 2021) suggests oral iron supplementation may benefit coronary heart disease patients with iron deficiency by improving exercise tolerance and quality of life. Conversely, for patients with TS > 30.5%, management should focus on reducing iron burden through dietary modifications, iron chelation therapy when indicated, or treating underlying iron metabolism disorders, while monitoring cardiovascular and hepatic functions to prevent iron toxicity complications. All interventions should be individualized with careful TS monitoring to maintain levels within the optimal 15–30% range. Patients outside this range should be considered high-risk and require more frequent follow-up assessment. Overall, our study provides novel insights into the complex, nonlinear relationship between TS and mortality, with a focus on the mediating role of inflammation at different TS levels. These findings emphasize the need for individualized management of TS based on its levels to minimize mortality risk in CAD patients. Future research should further explore the interplay between TS dynamics, systemic inflammation, and oxidative stress across diverse populations and disease conditions. In addition to its inflammatory effects, TS also influences cardiac function and congestion, further contributing to its prognostic significance in CAD. Low TS has been associated with impaired ventricular function, increased NT-proBNP levels, and greater congestion, all of which are predictors of poor outcomes in CAD patients [25,26]. At the same time, iron overload has been implicated in left ventricular dysfunction and arrhythmogenesis, highlighting the bidirectional risks of TS extremes. These findings underscore the importance of TS as a biomarker for assessing both systemic inflammation and cardiac-specific risks in CAD patients. Our study suggests that the association between low TS and mortality may be driven by chronic inflammation rather than simple iron deficiency. This finding provides critical clinical implications: for CAD patients with low TS, stratified management based on ferritin and inflammatory markers is essential. In patients with active inflammation, indiscriminate iron supplementation may fail to improve prognosis and could potentially exacerbate oxidative injury. Future studies should explore whether targeting hepcidin or anti-inflammatory therapies could disrupt the vicious cycle of inflammation and iron dysregulation [27].

There are several limitations in our study that should be acknowledged. First, while we identified a significant association between TS and coronary heart disease (CAD) all-cause mortality, TS was only measured at baseline, and we were unable to account for potential dynamic changes in TS over time, which may influence the observed outcomes. Second, our study lacked detailed information on participants’ medication history, which could have impacted TS levels and confounded the relationship between TS and CAD mortality. Third, it should be emphasized that iron metabolism regulation is complex, and a single TS indicator has significant limitations in clinical decision-making. While TS provides valuable insights into iron transport capacity, a comprehensive assessment of iron status requires integration of multiple biomarkers. Finally, as an observational study, our findings cannot establish causality between TS and CAD mortality, and residual confounding cannot be completely excluded. Further longitudinal and interventional studies are warranted to confirm our findings and elucidate the causal pathways involved.

## Conclusion

In conclusion, our study reveals a significant nonlinear, U-shaped relationship between transferrin saturation (TS) and all-cause mortality in patients with coronary artery disease (CAD), with an inflection point at 30.5%. TS levels below 30.5% were associated with increased mortality risk, mediated in part by systemic inflammation, while TS levels above this threshold were also linked to elevated mortality. These findings underscore the importance of maintaining TS within an optimal range (20–30%) to minimize mortality risk in CAD patients. Furthermore, the mediating role of systemic inflammation highlights the potential for targeted interventions addressing both iron metabolism and inflammatory pathways to improve outcomes in this high-risk population. Future studies should focus on the dynamic changes in TS, the interplay between TS, inflammation, and oxidative stress, and the long-term effects of TS modulation on mortality risk to refine individualized management strategies for CAD patients.

## Supporting information

S1 File Supplementary Table 1. Mediation effects of inflammatory markers (SIRI, SII, and MLR) on the association between low transferrin saturation (TS ≤30.5%) and all-cause mortality in CAD patients. Supplementary Table 2. Reverse mediation analysis examining the pathway of inflammatory markers on TS and mortality when TS ≤30.5% in CAD patients. Supplementary Table 3. Mediation effects of inflammatory markers (SIRI, SII, and MLR) on the association between high transferrin saturation (TS >30.5%) and all-cause mortality in CAD patients. (DOCX)
