# Supplementary material for: Mediating role of systemic inflammation in linking transferrin saturation to all-cause mortality in patients with coronary artery disease: Evidence from a large population-based study
Source: PLoS One. 2025 Jun 2;20(6):e0322633. doi: 10.1371/journal.pone.0322633 (PMC12129200; doi:10.1371/journal.pone.0322633)
Supplement: S1 File — (DOCX) [file pone.0322633.s001.docx]

**Supplement Table 1. Mediation effects of inflammatory markers on the association between TS (≤30.5%) and all-cause mortality.**

| **IRIs** | Estimate | 95% CI lower | 95% CI upper | P-value |
| --- | --- | --- | --- | --- |
| SIRI |  |  |  |  |
| Total effect | -0.074 | -0.135 | -0.013 | 0.016 |
| Mediation effect | -0.021 | -0.044 | -0.006 | <0.0001 |
| Direct effect | -0.053 | -0.111 | 0.006 | 0.084 |
| Proportion mediated | 0.285 | 0.069 | 1.147 | 0.016 |
| SII |  |  |  |  |
| Total effect | -0.074 | -0.133 | -0.012 | 0.018 |
| Mediation effect | -0.016 | -0.035 | -0.004 | 0.002 |
| Direct effect | -0.058 | -0.115 | 0.003 | 0.066 |
| Proportion mediated | 0.218 | 0.038 | 0.958 | 0.020 |
| MLR |  |  |  |  |
| Total effect | -0.073 | -0.131 | -0.012 | 0.020 |
| Mediation effect | -0.010 | -0.024 | 0.001 | 0.080 |
| Direct effect | -0.064 | -0.119 | -0.006 | 0.036 |
| Proportion mediated | 0.130 | -0.053 | 0.584 | 0.100 |
| NLR |  |  |  |  |
| Total effect | -0.075 | -0.135 | -0.012 | 0.018 |
| Mediation effect | -0.013 | -0.036 | 0.000 | 0.058 |
| Direct effect | -0.062 | -0.118 | -0.003 | 0.044 |
| Proportion mediated | 0.174 | -0.023 | 0.696 | 0.072 |
| PLR |  |  |  |  |
| Total effect | -0.073 | -0.129 | -0.011 | 0.018 |
| Mediation effect | -0.006 | -0.017 | 0.000 | 0.078 |
| Direct effect | -0.067 | -0.124 | -0.006 | 0.028 |
| Proportion mediated | 0.077 | -0.015 | 0.411 | 0.096 |
| NPR |  |  |  |  |
| Total effect | -0.074 | -0.134 | -0.014 | 0.016 |
| Mediation effect | -0.001 | -0.033 | 0.001 | 0.464 |
| Direct effect | -0.073 | -0.128 | -0.009 | 0.016 |
| Proportion mediated | 0.015 | -0.028 | 0.427 | 0.460 |

**Supplementary Table 2. Reverse Mediation Analysis: Effects of Inflammatory Markers on TS and Mortality when TS ≤30.5%**

| **IRIs** | **Proportion mediated effect** | **95% CI lower** | **95% CI upper** | **P-value** |
| --- | --- | --- | --- | --- |
| SIRI | 0.022 | -0.041 | 0.076 | 0.531 |
| SII | 0.011 | -0.067 | 0.091 | 0.729 |
| MLR | 0.034 | -0.014 | 0.087 | 0.223 |
| NLR | 0.013 | -0.039 | 0.057 | 0.549 |
| PLR | 0.032 | -0.041 | 0.112 | 0.300 |
| NPR | 0.015 | -0.587 | 0.534 | 0.812 |

**Supplementary Table 3. Mediation effects of inflammatory markers on the association between TS (>30.5%) and all-cause mortality in CAD**

| **IRIs** | Estimate | 95% CI lower | 95% CI upper | P-value |
| --- | --- | --- | --- | --- |
| SIRI |  |  |  |  |
| Total effect | 0.054 | -0.008 | 0.141 | 0.096 |
| Mediation effect | 0.001 | -0.005 | 0.006 | 0.782 |
| Direct effect | 0.054 | -0.008 | 0.141 | 0.100 |
| Proportion mediated | 0.013 | -0.223 | 0.197 | 0.806 |
| SII |  |  |  |  |
| Total effect | 0.056 | -0.014 | 0.145 | 0.116 |
| Mediation effect | -0.001 | -0.009 | 0.009 | 0.856 |
| Direct effect | 0.056 | -0.012 | 0.147 | 0.110 |
| Proportion mediated | -0.016 | -0.386 | 0.466 | 0.900 |
| MLR |  |  |  |  |
| Total effect | 0.056 | -0.013 | 0.146 | 0.110 |
| Mediation effect | -0.001 | -0.014 | 0.006 | 0.624 |
| Direct effect | 0.057 | -0.010 | 0.149 | 0.106 |
| Proportion mediated | -0.024 | -0.486 | 0.375 | 0.694 |
| NLR |  |  |  |  |
| Total effect | 0.055 | -0.014 | 0.146 | 0.114 |
| Mediation effect | 0.000 | -0.007 | 0.007 | 0.970 |
| Direct effect | 0.055 | -0.013 | 0.146 | 0.124 |
| Proportion mediated | 0.004 | -0.281 | 0.273 | 0.992 |
| PLR |  |  |  |  |
| Total effect | 0.055 | -0.014 | 0.146 | 0.118 |
| Mediation effect | 0.001 | -0.008 | 0.007 | 0.980 |
| Direct effect | 0.055 | -0.015 | 0.148 | 0.122 |
| Proportion mediated | 0.004 | -0.284 | 0.438 | 0.986 |
| NPR |  |  |  |  |
| Total effect | 0.054 | -0.015 | 0.146 | 0.120 |
| Mediation effect | -0.001 | -0.016 | 0.009 | 0.710 |
| Direct effect | 0.056 | -0.011 | 0.146 | 0.110 |
| Proportion mediated | -0.025 | -0.820 | 0.408 | 0.742 |
